# Human cancer databases (Review)

**DOI:** 10.3892/or.2014.3579

**Published:** 2014-10-31

**Authors:** ATHANASIA PAVLOPOULOU, DEMETRIOS A. SPANDIDOS, IOANNIS MICHALOPOULOS

**Affiliations:** 1 Center of Systems Biology, Biomedical Research Foundation, Academy of Athens, Athens 11527, Greece; 2 Laboratory of Clinical Virology, Medical School, University of Crete, Heraklion 71003, Crete, Greece

**Keywords:** cancer, databases, genomics, transcriptomics, proteomics, epigenomics, immunomics, data mining

## Abstract

Cancer is one of the four major non-communicable diseases (NCD), responsible for ~14.6% of all human deaths. Currently, there are >100 different known types of cancer and >500 genes involved in cancer. Ongoing research efforts have been focused on cancer etiology and therapy. As a result, there is an exponential growth of cancer-associated data from diverse resources, such as scientific publications, genome-wide association studies, gene expression experiments, gene-gene or protein-protein interaction data, enzymatic assays, epigenomics, immunomics and cytogenetics, stored in relevant repositories. These data are complex and heterogeneous, ranging from unprocessed, unstructured data in the form of raw sequences and polymorphisms to well-annotated, structured data. Consequently, the storage, mining, retrieval and analysis of these data in an efficient and meaningful manner pose a major challenge to biomedical investigators. In the current review, we present the central, publicly accessible databases that contain data pertinent to cancer, the resources available for delivering and analyzing information from these databases, as well as databases dedicated to specific types of cancer. Examples for this wealth of cancer-related information and bioinformatic tools have also been provided.

## 1. Comprehensive cancer projects

Large-scale collaborative cancer projects generate a large amount of cancer data. The International Cancer Genome Consortium (ICGC) (1) and The Cancer Genome Atlas (TCGA) (2) are the most prominent examples of such efforts (Table I). ICGC aims to obtain a comprehensive description of the genomic, transcriptomic and epigenomic changes in 50 different tumor types and/or subtypes that are of clinical and social significance (1). The curated data types are: sample donor IDs, cancer project, simple somatic mutations (SSMs) and genes with SSMs. The data are complemented by associated attributes, including the primary site of the tumor at diagnosis (e.g., brain, skin, blood, bone and prostate), gender, tumor stage at diagnosis (e.g., 4, M0, M3, 1, M1), available data types (e.g., copy number and structural somatic mutations, miRNA expression, gene expression, DNA methylation and exon junction.

The ICGC Data Portal (3) provides tools for querying, visualizing and downloading the data released quarterly by the consortium’s member projects. The ICGC Data Portal contains data from other large-scale cancer genome projects, including TCGA, Johns Hopkins University (Baltimore, MD, USA) (4,5) and Tumor Sequencing Project (TSP) (6). The ICGC Data Portal is based on the BioMart data management platform (7,8), which uses a seamless federated data model to enable the cross querying of diverse biological databases in a unified manner. To maintain the uniformity of ICGC datasets, the same set of data models, ontologies, controlled vocabularies and references have been applied in all of the ICGC’s member databases. Three interfaces are available: cancer projects, advanced search and data repository. The cancer projects interface contains data available in the 49 ICGC member projects, as well as additional filters and a selection of attributes. The advanced search interface contains the complete set of filters and attributes. The database can be queried interactively using three main options: donors, genes and mutations. By selecting any of these options, the results are presented in tabulated form. The results can be filtered based on several search criteria. The Data Repository provides access to all ICGC cancer project data, including uniformly processed and annotated data files. The results can be downloaded and exported for further analysis.

**Figure 1:**
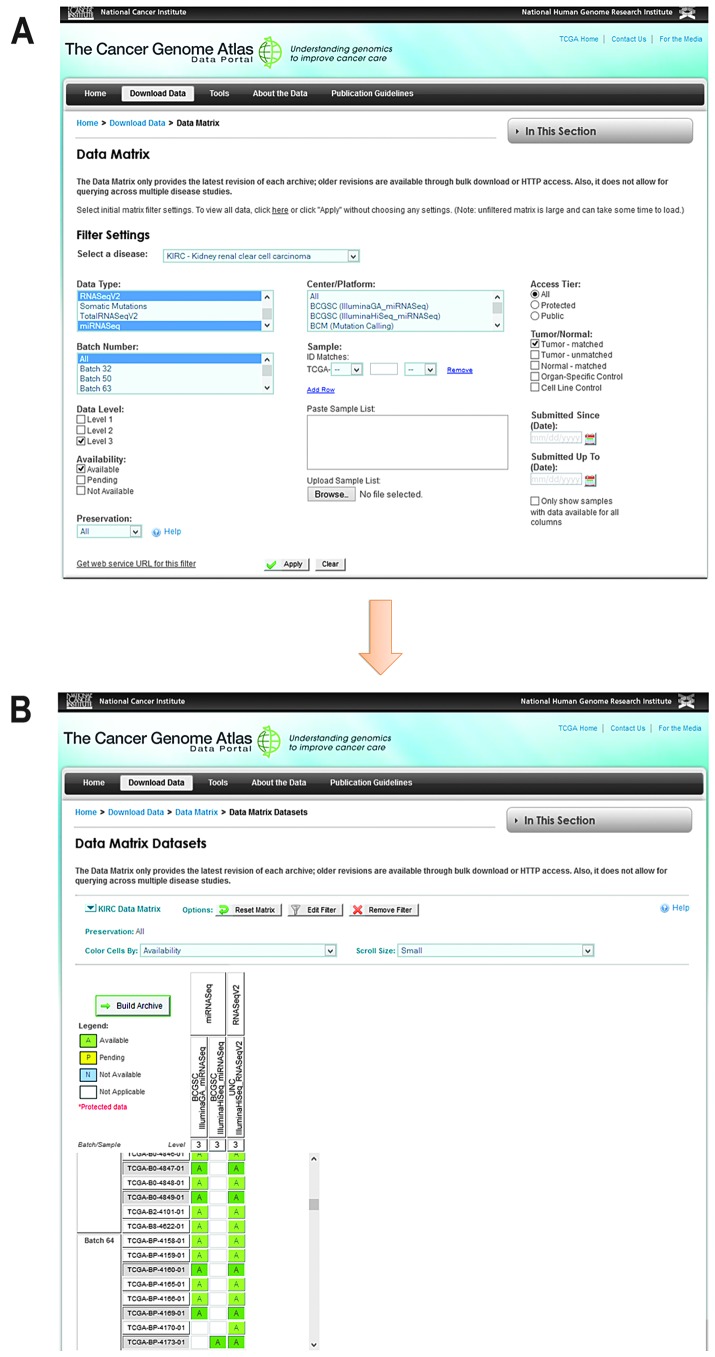
Example of searching for and downloading data from The Cancer Genome Atlas (TCGA). (A) Attributes are selected and (B) the results are presented on a different page. The user can select samples by clicking on them (shadowed). The data can be downloaded by clicking on the ‘Build Archive’ button.

TCGA is a joint project of the National Cancer Institute (NCI) and the National Human Genome Research Institute (NHGRI) (both from Bethesda, MD, USA) that provides a comprehensive map of the important genomic changes that occur in the major types and subtypes of cancer (2). It contains clinical information, genomic characterization data and high level sequence analysis of the tumor genomes. The TCGA Data Portal enables investigators to explore, download and analyze datasets generated by TCGA. The data types stored in TCGA include gene expression, copy number, somatic mutations, single nucleotide polymorphisms (SNPs), microRNAs, clinical outcomes and tissue slide images. Four main methods for downloading data are available: i) Data Matrix enables users to select and download a subset of data for a particular cancer type, but does not allow searching and downloading data across multiple cancer types simultaneously; ii) Bulk Download facilitates the bulk download of archives of data as uploaded by the TCGA Centers; iii) File Search allows users to filter and download data files in a more easily accessible manner; and iv) Access HTTP Directories enables users to access the HTTP directories where the data archives are stored. The TCGA Roadmap (9) engine was developed to index and annotate the TCGA files and capture file metadata in the TCGA open-access HTTP by applying third-generation web technologies (Web 3.0) (10). An example of searching for and downloading processed data concerning expressed genes and microRNAs from next generation sequencing (NGS) experiments in matched tumors is shown in Fig. 1.

The Clinical Proteomic Tumor Analysis Consortium (CPTAC) (11,12), launched by NCI, aims at elucidating the molecular basis of cancer through the application of proteomic techniques. In particular, CPTAC analyzes cancer samples by mass spectrometry in order to identify and quantify their constituent proteins and localize the post-translational protein modifications, such as phosphorylation. The CPTAC Data Portal is the central repository for the distribution of proteomic data collected by the Proteome Characterization Centers (PCCs). The CPTAC Data Portal uses an Aspera Connect Transfer Server for the transport of large data files. The Cancer Genome Project (CGP) (13) at the Sanger Institute (Cambridge, UK), seeks to identify somatic variants/mutations critical in the etiology and pathogenesis of human cancers by using the sequenced human genome and high-throughput mutation detection technologies.

## 2. Resources

The large volume of data that emerges from these large-scale programs has resulted in the concomitant development of new databases for accessing and analyzing cancer data (Table I).

### Tools

Specialized web-based tools are available to enable investigators to query, retrieve and analyze cancer-related data in a rapid, reliable and efficient manner. The Cancer Genome Anatomy Project (CGAP) (14) of NCI includes a number of bioinformatic analysis tools and interconnected modules that enable users to access CGAP data. These data include cancer-relevant genes and SNPs, malignant tissues and chromosomal aberrations in cancer patients. Moreover, CGAP provides information regarding the differential expression of a given gene in normal, precancerous and cancerous tissues based on Serial Analysis of Gene Expression (SAGE), as well as RNA interference (RNAi) constructs that target cancer-related genes, and diagrams of biochemical pathways and protein complexes. The UCSC Cancer Genomics Browser (15,16) is a suite of web-based tools used to integrate, display and analyze cancer genomics and clinical data. The browser allows whole-genome views of several different types of genomics and associated clinical data. Various datasets can be viewed together as coordinated ‘heatmap tracks’, thus enabling the user to make comparisons across studies and cancer types. Annotated biological pathways, collections of genes, genomic or clinical information can be sorted, filtered, aggregated, classified and viewed interactively based on any given feature set, including clinical features, annotated biological pathways and user-contributed collections of genes. The Cancer Genome Workbench (CGWB) (17) includes copy number, mutation, expression and methylation data from various projects, including TCGA, the Catalogue of Somatic Mutations in Cancer (COSMIC) (18,19), Johns Hopkins University, and the Therapeutically Applicable Research To Generate Effective Treatments (TARGET) initiative. CGWB provides a series of tools for visualizing genomic and transcription alterations from different cancer samples. The data in CGWB can be viewed in three different ways: i) Integrated track, which provides a sample-level view of genomic alterations from multiple data sources; ii) Heatmap view, an interactive graphical view of gene expression and copy number data and their associated clinical features; and iii) Bambino, an alignment viewer for NGS data.

### Cancer driver genes

Several repositories of driver genes or gene families that play a causal role in carcinogenesis have been developed. The Tumor Gene Family Databases (TGDBs) (20) contain a broad range of information regarding genes involved in cancer. Apart from TGDB itself, the data of two component databases, the Oral Cancer Gene Database (OrCGDB) (21) and the Breast Cancer Gene Database (BCGD) (22), have been merged into TGDBs. Gene information includes gene aliases, cell location, biochemical function, frequency in various tumors, oncogenicity, chromosomal location, tumor gene type (either proto-oncogene or tumor suppressor gene) and the signal transduction pathways in which the gene of interest is involved.

The DriverDB database (23) compiles a large amount (>6,000 cases) of exome-sequencing (exome-seq) data, annotation databases such as dbSNP (24), 1000 Genome (25) and COSMIC, as well as various bioinformatics algorithms for the identification of driver genes or mutations. The database can be queried either by cancer type, where the driver genes/mutations for a specific cancer type are estimated, or by gene where the mutation information of a driver gene in five different aspects is presented. Meta-Analysis, another option offered in DriverDB, enables users to identify driver genes in custom-defined samples according to clinical criteria. The RAS Oncogene Database (RASOnD) (20) integrates large amounts of genomics and proteomics data derived from publicly available databases such as NCBI’s GenBank (26), Online Mendelian Inheritance in Man (OMIM) (27), Universal Protein Resource (UniProt) (28), Protein Databank (PDB) (29), Kyoto Encyclopedia of Genes and Genomes (KEGG) (30) and PubMed (31). The RASOnD database contains 199,046 entries from 101 species, allowing investigators to retrieve information regarding RAS oncogene SNPs, chromosomal positions, disease associations and nucleotide and amino acid positions.

### Genetic variations

**Figure 2:**
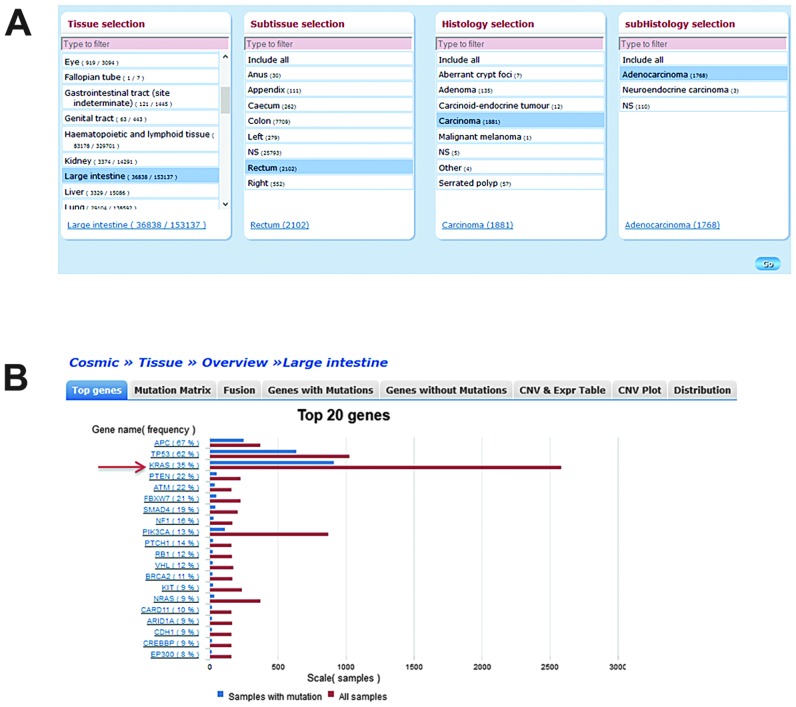

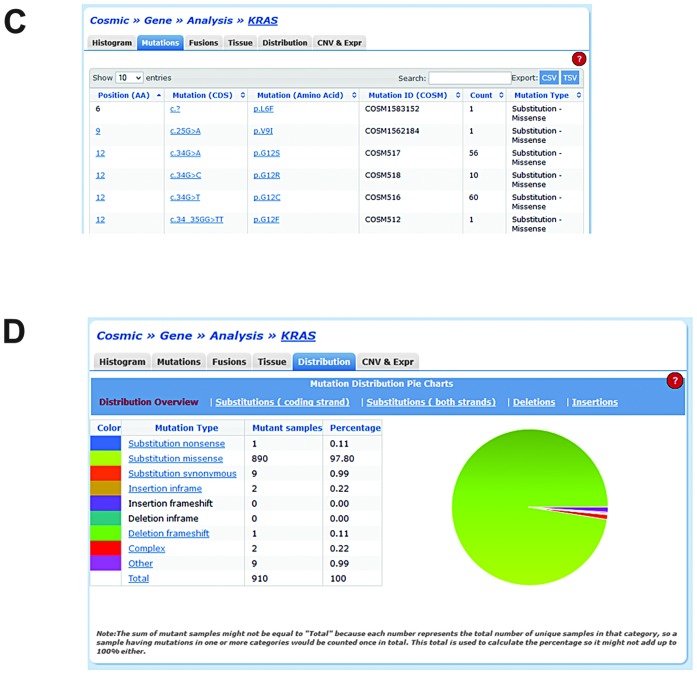
Example of querying COSMIC database. (A) Cancer attributes are selected. (B) A list of the top 20 genes involved in the particular type of cancer. (C) A list of the mutations along with links and pertinent information and (D) distribution of the mutations present in the *KRAS* gene.

Cancer is characterized by abundant genetic abnormalities in the form of mutations, SNPs, copy number alterations (CNAs), genomic rearrangements and gene fusions. To manage the increasing amount of information, public resources have been implemented to collect, curate, annotate and analyze data regarding cancer genetic variations. COSMIC (18,19) is the largest public database that stores and displays information on somatically acquired mutations involved in cancer and associated clinical and phenotypic data. Currently, COSMIC contains information on 28,735 genes, 2,002,811 coding mutations and 10,435 fusion gene mutations reported in 1,029,547 cancer samples. The data are primarily extracted from published scientific literature and whole-genome sequencing screens from CGP. To provide a uniform representation of the data, a histology and tissue ontology has been created. COSMIC uses the BioMart (32) data mining software that enables users to filter the available data according to cancer sample, gene, mutation, tumor site, histology and tumor. The results are presented in tabulated format (Fig. 2).

The Cancer Gene Census (CGC) (33) lists >1% of all human genes which bear mutations that causally contribute to carcinogenesis. The Gene Census data include gene symbol according to HGNC (34), a short description of the gene, gene chromosomal location, type of mutations (i.e., somatic or germline), type of tumor and the cancer syndrome in which the mutated gene is involved. The data are provided in a table and can be downloaded and exported in several formats. The catalogue is updated at regular intervals. BioMuta (35) is a curated database of cancer-related non-synonymous single-nucleotide variations (nsSNVs) that affect functional sites. The datasets are derived from the TCGA, COSMIC, ClinVar (36) and UniProt Knowledgease (UniProtKB) (37) databases. Due to the large amount of data present in the primary NGS repositories, the High-performance Integrated Virtual Environment (HIVE) platform (35) has been implemented in BioMuta in order to store, analyze, compute and curate NGS data and associated metadata. CaSNP (38) is a comprehensive collection of CNAs from 11,485 Affymetrix SNP arrays with raw data from NCBI’s Gene Expression Omnibus (GEO) (39), additional arrays from the TCGA consortium and a few individual publications covering 34 different cancer types in 105 studies. The user can query CaSNP by gene, region or cancer type and retrieve information regarding the frequencies of copy number aberrations for each study. CaSNP also provides a heatmap showing CNAs estimated at each SNP marker around the query region across all studies. CanProVar (40) has been developed to store and prevent germline and somatic amino acid variations in the human proteome associated with human tumorigenesis based on published literature. The CanGEM (41) database stores clinical information on tumor samples and array Comparative Genomic Hybridization (aCGH) data to detect gene CNAs in cancer. Users can create custom datasets for specific clinical sample characteristics or CNAs of individual genes. The Integrative Cancer Profiler System (ICPS) (10) database integrates genomic alterations such asCNA and LOH, with transcription signatures (SAGE, microarray) in order to study gene profiles in one or more different types/subtypes of cancer. Currently, ICPS contains five different data types and 23,375 experiments covering 11 major cancer types. Apart from public data, ICPS also supports in-house data of users.

Given that ~50% of human tumors harbor TP53 gene mutations (42), a UMD TP53 (43) database was created to provide detailed information on TP53 mutants such as the molecular and cell properties of each TP53 mutant and localization or various gains of functions. The UMD TP53 database contains >110,000 entries. The International Agency for Research on Cancer (IARC) TP53 (44,45) is a comprehensive resource that compiles all TP53 gene variations in human cancers derived from scientific publications. The datasets available in the resource are: TP53 somatic and germline mutations, validated common TP53 polymorphisms identified in human population and their functional and clinical impact, TP53 gene status (i.e., wild-type, mutant, null) in various human cell lines, mouse models with engineered TP53 constructs and experimentally induced TP53 mutations.

### Epigenetic modifications

Epigenetic modifications, such as DNA methylation and chromatin-modifying factors, play a critical role in carcinogenesis by regulating tumor-suppressor gene silencing, proto-oncogene activation and chromosomal instability (46). MethyCancer (47), the database of human DNA methylation and cancer, was developed to study the association of DNA methylation, gene expression and cancer. It contains data of DNA methylation, cancer-relevant genes and CpG Island (CGI) clones derived from high-throughput sequencing. The MethyView option allows the graphical presentation of CGI information of >30,000 genes. PubMeth (48) is a cancer methylation database that includes information of genes that have been reported in the literature to be methylated in various cancer types. The information is extracted from PubMed abstracts using a text-mining approach, GoldMine, followed by manual annotation. There are two options for searching the database, the ‘gene-centric’ (the cancer types/subtypes where the genes of the interest are reported to be methylated) and the ‘cancer-centric’ (the genes reported to be methylated in a particular cancer type/subtype). In ChromoHub V2 (49), chemical, structural and biological data extracted from public repositories, such as TCGA and ICGC, are mapped on phylogenetic trees of protein families involved in chromatin-mediated signaling.

### OncomiRs

OncomiRs, microRNAs that are associated with diverse cancer-related processes, play a significant role in the epigenetic regulation of cancer. The miRCancer database (50) provides a comprehensive collection of microRNA expression profiles in various human malignancies that are automatically extracted from publications in PubMed. OncomiRDB (51) is a database developed for annotating the experimentally validated oncomiRs from literature. The database includes 2,259 entries of oncomiR regulations, covering 328 miRNAs and 829 target genes in 25 cancer tissues extracted from published literature. The user is able to search by miRNA, tissue, tumor, target gene and function (e.g., proliferation, apoptosis, migration).

### Transcriptomics

**Figure 3:**
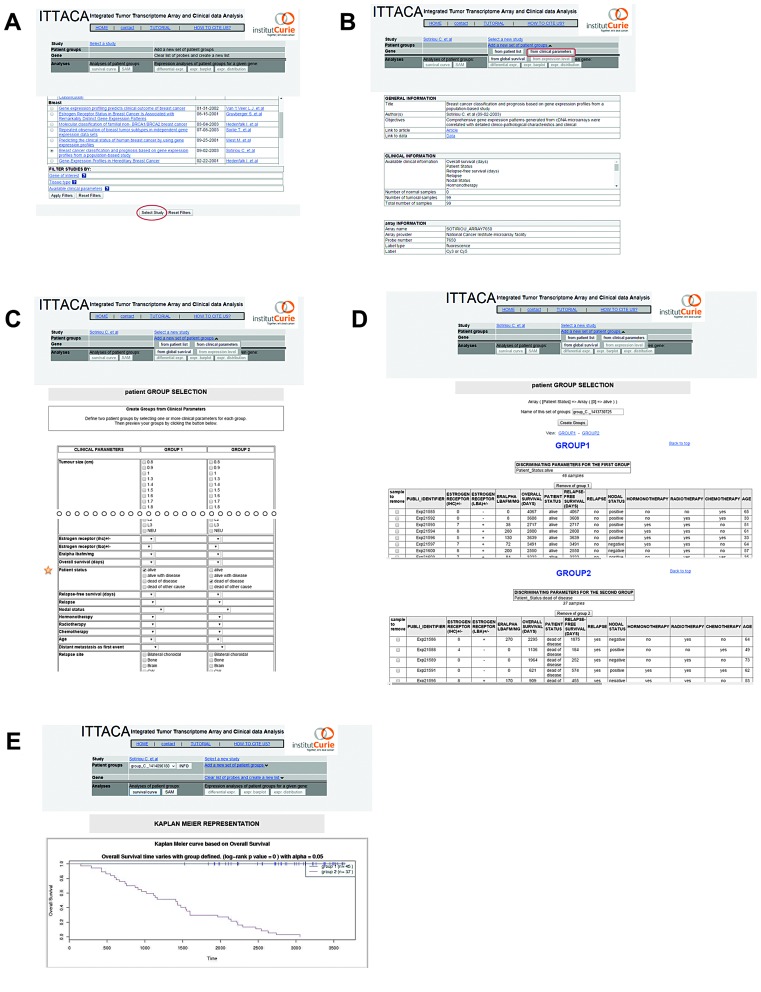
Example of querying Integrated Tumor Transcriptome Array and Clinical data Analysis (ITTACA) database. (A) A study was selected and (B) a new set of patient groups from clinical parameters was added. In a new page, (C) the values ‘alive’ and ‘dead of disease’ under the clinical parameter ‘Patient status’ were chosen. (D) Two different groups corresponding to the two different patient statuses were evident. The patient groups were subsequently analyzed using a survival curve. (E) A Kaplan-Meier curve based on overall survival was generated.

Databases have been designated to extract, store and interpret data from large-scale and genome-wide expression studies. Oncomine (52) is a cancer microarray database that collects and curates 715 gene expression data-sets and 86,733 samples and associated clinical data from most major types of data. Oncomine allows a user to contact a gene-centric search to retrieve the differential expression analyses of a gene of interest across all available datasets; in a study-centric search, the genes that are differentially expressed in the selected study are provided. To facilitate data mining, the current version of Oncomine enables multi-gene search, gene ontology-based filtering and integration of Oncomine concepts. Integrated Tumor Transcriptome Array and Clinical data Analysis (ITTACA) (53) is a central repository of transcriptome microarray and associated clinical data from breast carcinoma, bladder carcinoma and uveal melanoma. A web interface offers different options for class comparison analyses, such as the comparison of profiles of expression distribution and patient survival analyses. The user is able to analyze the differential expression of one or more gene between two groups of samples with different phenotypes, and, conversely, the genes differentially expressed between two groups of samples (Fig. 3).

The Cancer Gene Expression Database (CGED) (54) contains cancer gene expression profiles and related clinical information. The expression data are obtained by adaptor-tagged competitive PCR from breast, colorectal, esophageal, gastric, hepatocellular, lung and thyroid cancers and glioma. The database can be queried either using gene identifiers or by functional categories. Mosaic plots are used for the visualization of gene expression data and comparison of the expression patterns of various genes. CancerMA (55) is an integrated bioinformatic pipeline used for the automated identification of novel candidate cancer biomarkers by analyzing the expression profiles of a user-defined gene list across public cancer microarray [GEO, ArrayExpress (56)] experimentally verified datasets. A total of 80 microarray datasets covering 13 types of cancer are available.

### Proteins

**Figure 4:**
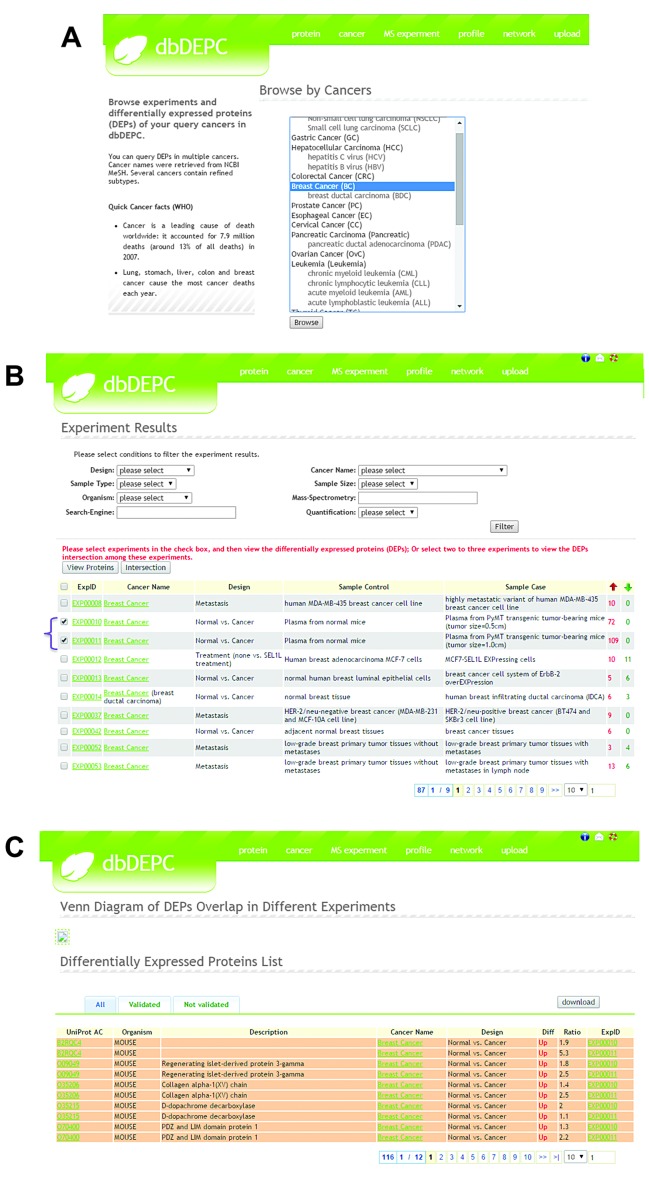
Screenshots showing (A) the results of selecting Breast Cancer (BC) as query. (B) A table is provided where the differentially expressed proteins (DEPs) in BC are shown, including pertinent information and links. By selecting two experiments, (C) the results are returned in a tabulated form where the DEPs in the two experiments are shown. There are links to Universal Protein Resource (UniProt) and back to the experiments.

Differentially expressed proteins (DEPs) that contribute to the onset and progression of cancer have been identified. The first database of DEPs in human cancers, dbDEPC (57,58), currently contains 4,029 DEPs, curated from 331 mass spectrometry experiments across 20 types of human cancer. This resource enables the users to investigate whether a protein of interest has altered in particular cancers and, to create an association network of query proteins. Moreover, dbDEPC shows a heatmap that represents the expression profiles of a certain protein across various cancer types. An example of how to query dbDEPC is shown in Fig. 4.

### Phosphorylation

The genes encoding protein kinases, enzymes that phosphorylate proteins, are among the most commonly mutated genes in human cancers. The MoKCa database (59) provides a collection of the mutations present in protein kinases involved in cancer, along with structural and functional annotation and, wherever possible, prediction of the impact of these mutations in the structure and function of kinases. The user can select from a pull-down list the gene that codes for a protein kinase: information is available for the types of mutations (e.g., missense, silent) found in tumor cell lines and, the mutated amino acid residues are mapped onto the tertiary structures of the affected protein kinase domains.

### Cell lines

Cancers are thought to be initiated and maintained by a subpopulation of stem or stem-like cells with tumorigenic potential (60). SCDE (61,62) is an integrated repository of curated tissue and cancer stem cell data from blood, brain and intestine. The datasets are homogenized with regard to structure, formatting and annotation and stored in the Investigation/Study/Assay-Tab (ISA-Tab) format. SCDE is linked to the Galaxy framework which provides a series of analytical tools to compare those data to genes, molecular signatures and pathways. The CellLineNavigator (63) database contains gene expression profiles (generated uniformly) of >300 human cancer lines. On the basis of their phenotypic attributes, these cell lines were further classified into 28 tissues of origin and 57 different disease states. The database is also linked to advanced tools of bioinformatics analyses. The database can be searched for i) differentially expressed genes; ii) pathological or physiological states; and iii) gene names or functional characteristics, such as Gene Ontologies (GOs) (64) and KEGG pathway maps. A combination of all query options is also possible.

### Cytogenetics

Chromosomal aberrations, such as translocations and their corresponding gene fusions and duplications or deletions and generated gene gains or losses are known to play an important role in the onset of tumorigenesis (65). The Mitelman Database of Chromosome Aberrations and Gene Fusions in Cancer (65) is a repository of chromosomal rearrangements such as translocations and their resulting gene fusions that are associated with tumor characteristics. The data contained in the database have been manually extracted from literature. The current version of the database includes a total of 64,679 cases and 2,094 gene fusions. The Mitelman Database can be queried by several different search options. In particular, the user can search for individual patient cases or associations of cases, such as concurrent chromosomal aberrations and clinical associations of cytogenetic abnormalities, or references themselves.

### Immunomics

Tumor-associated antigens (TAAs) have been applied extensively in the clinical diagnosis and treatment of human cancers. The publicly available Human Potential Tumor Associated Antigen (HPtaa) (66) database contains potential TTAs identified by *in silico* computing. HPtaa incorporates publicly available microarray expression data, GEO’s SAGE data and Unigene (67) expression data, as well as other relevant knowledge bases such as CGAP. Currently, a total of 3,518 potential targets are included in the database. A web query interface enables users to search for potential TAAs overexpressed in several cancer types with particular gene features including chromosome (X and Y or euchromosome), coding capacity (protein-coding genes or else) and subcellular location (membrane or secretory proteins). The human immune response (humoral and cellular) to an increasing number of TAAs has also been well documented. The Academy of Cancer Immunology supported by the Ludwig Institute (both from New York, NY, USA) for Cancer Research have established the Cancer Immunome Database (CID) (68) which provides information on all the gene products against which an immunome response has been reported in cancer patients. The user can access information regarding the genes that encode the cancer antigens. Given that a gene can yield multiple antigenic epitopes, the frequency with which the antigenic epitopes are recognized by sera or cells from healthy and diseased individuals is reported. Wherever appropriate, access is provided to experimental evidence (serological results, microscope images and cytotoxic assays) or patients’ information (the type of cancer from which they are suffering, the disease stage, and time the samples were obtained). CTdatabase (69) is a curated repository of annotated and computationally predicted cancer-testis (CT) antigens. The CT antigens are broadly classified according to their expression pattern in human healthy tissue. CTdatabase also provides information on genes, the verified splice variants, genomic locations, gene duplications and bibliographical references.

### Anticancer agents

Knowledge bases dedicated to cancer translational research and identification of drugs and compounds that inhibit cancer-related target genes are also available. CanSAR (70) is a public resource that supports cancer translational research and finding of drug through the integration of biological, chemical, pharmacological and disease data, structural biology and cellular networks. The user, through a single portal, is able to access information regarding genes, protein families, cell lines and compounds, as well as approved drugs and clinical candidates associates with cancer. CancerResource (71) is a comprehensive knowledge base that integrates cancer-relevant relationships of compounds/drugs and targets deduced from the text mining of >19 million PubMed abstracts and external resources such as Therapeutic Target Database (TTD) (72), Comparative Toxicogenomics Database (CTD) (73), Pharmacogenomics Knowledge Base (PharmGKB) (74) and DrugBank (75). CancerResource can be queried by Cancer (the user can view the genes expressed in specific cancer tissues, and also browse cancer-related KEGG pathways), Drug (the user can search by compound or drug and obtain information about the cancer relevance of the query drug/compound and its interactions with targets) and Target (drugs interacting with targets). The Anticancer Agent Mechanism Database (76,77) contains a list of 122 compounds with anticancer activity classified by their mechanism of action into alkylating agents, topoisomerase I/II inhibitors, RNA/DNA antimetabolites, and antimitotic agents. This set is generated by neural networks able to predict the mechanism of action of a drug based on its pattern of activity against a diverse panel of human tumor cell lines in the NCI drug screening program.

### Drug resistance

A major obstacle in cancer therapies is the development of drug resistance based on mutations in drug targets. Therefore, it is important to identify mutations in drug targets responsible for drug resistance. CancerDR (78) provides information of 148 anticancer drugs and their pharmacological profiling across ~1,000 cancer cell lines. Pharmacological profiling information of these anticancer drugs was collected from the Cancer Cell Line Encyclopedia (CCLE) (79) and COSMIC databases. CancerDR provides information about each drug target (cancer genes) that corresponds to these anti-cancer drugs, such as gene sequences in respective cancer cell lines, mutations, function and structure. This database allows users to search for drug targets, drugs, cell lines and structure. Clustering of cell lines on the basis of their drug sensitivity towards a drug target allows users to identify groups of cell lines, which are resistance to a particular anticancer drug, as well as multipotent drugs effective against a wide range of cancer cell lines. The clustering of sequences of a drug target is important to identify mutants/variants against the corresponding drug target.

### Integrative resources

**Figure 5:**
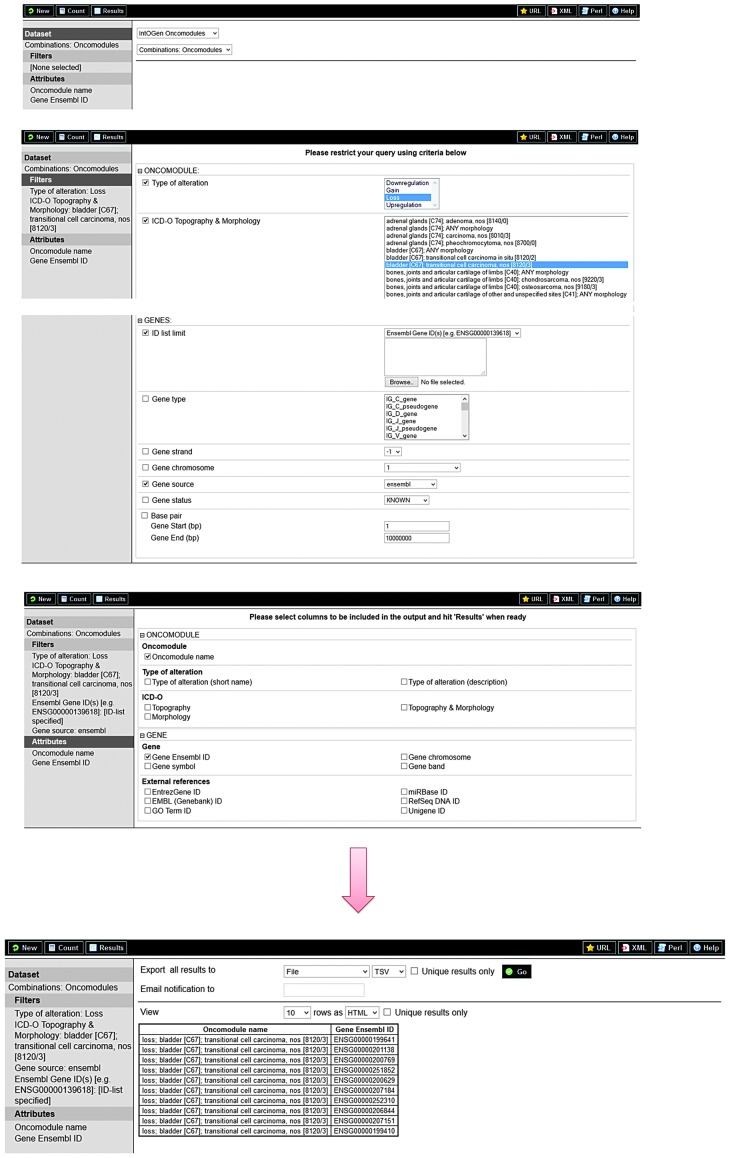
Example of querying IntOGen for the genes lost in a type of bladder cancer. Dataset, Filters and Attributes were selected. By clicking on the ‘Results’ button, the results are returned and can be viewed and exported in several formats.

IntOGen (80) is an integrative resource of high-throughput data associated with genomic, transcriptional, mutational alterations and modules (e.g., GO terms, KEGG pathways) involved in carcinogenesis. IntOGen collects data from various resources such as COSMIC, GEO, ArrayExpress, Progenetix (81), TCGA and CGP. Tumor samples in IntOGen are annotated with terms from the International Classification of Diseases for Oncology (ICD-O) (82) where the tumors are classified based on their topography (location in the human body) and histology (morphology). IntOGen can be queried by Genes, Projects, Cancer sites and Pathways. The Biomart portal (83) enables more complicated queries and the bulk download of all analysis results. The interface has a number of filters and attributes. IntOGen Biomart can be queried based on i) IntOGen Experiments, where the user can query gene or module (e.g., GO terms, KEGG pathways) information; ii) IntOGen Combinations, where the user is allowed to query a combination of experiments annotated with the same ICD-O term; and iii) IntOGenOncomodules which enables the user to search for combinations and experiments (Fig. 5). NCG 4.0 (84,85) is the current version of the Network of Cancer Genes, a repository of systems-level properties of cancer genes and oncomiRs (cancer-related microRNAs). It compiles information on 2,000 cancer genes that have been reported in literature to be mutated in 23 different types of cancer collected from 3,460 whole-exome and -genome screenings of cancer samples. NCG 4.0 reports information on the duplicability, functional annotation, evolutionary origin and interactions with other human proteins and microRNAs.

## 3. Cancer type-specific databases

Databases that focus on certain types or subtypes of cancer are also available (Table I).

**Figure 6:**
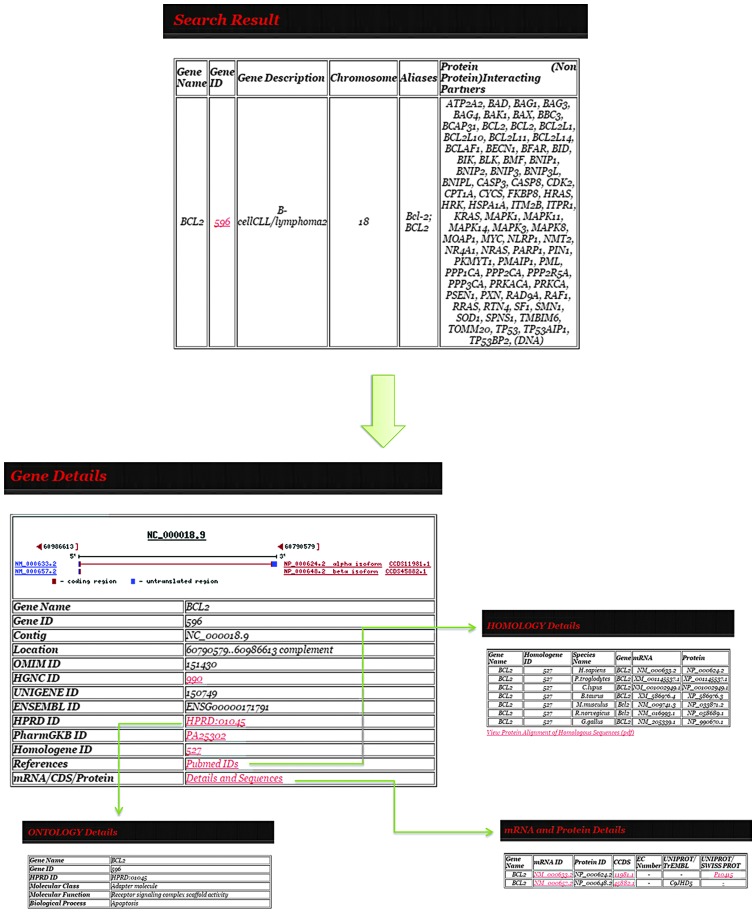
Results of a gene-centered search of Cervical Cancer gene DataBase (CCDB) using *BCL2* as query. Information is provided regarding gene ID, gene description, synonyms, chromosomal location and the molecules with which the gene interacts. There are also links to mRNA/CCDS/Protein sequence entries, to homologous genes from various species and gene ontology information.

The Cervical Cancer gene DataBase (CCDB) (86) contains a manually curated list of experimentally validated genes reported to be involved in different aspects of cervical carcinogenesis. Each record includes information concerning the gene of interest such as gene structure, chromosomal location, homology, ontology, and mRNA/CDC/protein sequences for each isoform encoded by the gene, as well as links to the original PubMed references and external databases [e.g., HGNC, Human Protein Reference Database (HPRD) (87), Homologene (67), PharmGKB, PDB]. The database can be queried by i) Gene name (the user can obtain information pertinent to the query gene); ii) Category, where the genes are grouped into categories; and iii) Chromosome number to view all cervical cancer-related genes present in a particular chromosome (Fig. 6).

The Dragon Database of Genes associated with Prostate Cancer (DDPC) (88) is an integrated resource of genes that have been experimentally confirmed to be involved in Prostate cancer. DDPC provides information about each gene such as experimental evidence, associated pathways, orthologous genes, gene ontologies, and related proteins. The user can select a gene from a pull-down list or search the database for genes using a combination of one or more options, including Anatomical System, Cell Line, KEGG Pathways, and Gene Ontology. DDPC also contains a list of the predicted transcription factor-binding sites on the promoters of genes included in the database. Moreover, the database contains DrugBank drugs reported to be associated with prostate cancer.

The curatedOvarianData (89) resource provides gene expression data and documented clinical annotations from 2,970 ovarian cancer patients from 23 studies with ovarian cancer across 11 microarray platforms. The data are made available as ExpressionSet objects for R/Bioconductor (90). The gene expression datasets are obtained from public databases, processed in a uniform manner and mapped to standard HGNC gene symbols (34).

The Genes-to-Systems Breast Cancer (G2SBC) Database (91) is an integrated resource of genes, transcripts and proteins reported in the literature to be dysregulated in breast cancer. Moreover, in G2SBC, the analysis is performed at different levels: the molecular components level, where the analysis is performed at the level of genes, transcripts and proteins; the molecular systems level, an analysis based on biological processes and protein-protein interaction networks; and the cellular systems level where the user can browse and simulate mathematical models of carcinogenesis, tumor growth and response to treatments. An ontology-based query system is also available for annotations associated with particular ontologies.

The HLungDB (92) is an integrated resource of lung cancer-related genes, proteins and microRNAs and pertinent clinical information extracted manually from the scientific literature. Each entry in the database describes the relationships between genes and lung cancer, containing detailed information of the gene, the expression pattern of the relevant gene (up- or downregulated) in the patient, experimentally verified information (e.g., transcription factor binding sites in the promoter of the gene) and protein-protein interaction networks. The database includes miRNAs that are differentially expressed in lung cancer or reported to be associated with lung cancer along with their experimentally verified identified targets. HLungDB is cross-linked to relevant external resources, including PubMed, HPRD, HUGO, IPI, EBI and KEGG. The lung cancer-related genes can be viewed either from a pull-down list where the genes are sorted by alphabetical order or by chromosome where the user can view all cancer-related genes located in the selected chromosome.

The Osteosarcoma Database (93) is a repository of osteosarcoma (OS)-relevant genes and microRNAs. The data stored in database are extracted from PubMed using an automated dictionary-based gene and microRNA recognition procedure, manual review and annotation. Currently, the database contains 911 protein-coding genes and 81 microRNAs deduced from 1,331 abstracts. The user is able to search by Gene or microRNA. Each entry is linked to PubMed.

The Pancreatic Expression Database (PED) (94), powered by the BioMart software, is a comprehensive resource of pancreatic cancer data from the literature obtained using a range of technologies, including genomics, transcriptomics, proteomics and miRNA. PED includes tools for mining data by using a combination of queries (e.g., gene expression and CNAs). The use of BioMart facilitates interoperability with other BioMart-compliant cancer resources, which allows users to expand their investigations to a number of relevant resources, such as Reactome, PRIDE and COSMIC.

The Renal Cancer Gene Database (RCDB) (95) is a manually curated repository of protein-coding genes and miRNAs associated with various forms of renal cell carcinomas (RCC). The protein-coding genes have been classified into six categories according to the type of alteration observed in RCC: i) methylation; ii) overexpression; iii) downregulation; iv) mutation; v) translocation; and vi) unclassified. RCDB also includes the miRNAs dysregulated in RCC. Users are able to query the protein-coding genes and miRNAs using keyword, category or, in the case of genes, chromosome. The ViroBLAST (96) tool is used to query a user-defined sequence against the sequences available in RCDB.

## Figures and Tables

**Table I:** Cancer databases.

Database	Description	URL	Refs.
Comprehensive cancer projects			
CGP	Cancer Genome Project	http://www.sanger.ac.uk/research/projects/cancergenome/	(13)
CPTAC	Clinical Proteomic Tumor Analysis Consortium	http://proteomics.cancer.gov/programs/cptacnetwork	(11,12)
ICGC	International Cancer Genome Consortium	https://www.icgc.org/	(1)
TCGA	The Cancer Genome Atlas	http://cancergenome.nih.gov/	(2)
Resources			
BioMuta	A framework for organizing cancer-related variations	https://hive.biochemistry.gwu.edu/tools/biomuta/biomuta.php	(35)
CancerDR	Cancer Drug Resistance Database	http://crdd.osdd.net/raghava/cancerdr/	(78)
CancerMA	An integrated bioinformatic pipeline for automated meta-analysis of public cancer microarray data	http://www.cancerma.org.uk/information.html	(55)
CancerResource	A resource of cancer-relevant compound and protein interactions	http://bioinf-data.charite.de/cancerresource/	(71)
CanGEM	Cancer Genome Mine	http://www.cangem.org/	(41)
CanProVar	Human Cancer Proteome Variation Database	http://bioinfo.vanderbilt.edu/canprovar/	(40)
CanSAR	Integrated Cancer Drug Discovery Platform	https://cansar.icr.ac.uk/	(70)
CaSNP	Cancer SNP data on CNAs	http://cistrome.dfci.harvard.edu/CaSNP/	(38)
CellLineNavigator	A web-based compendium of cancer cell line expression profiles	http://www.medicalgenomics.org/celllinenavigator/	(63)
CGAP	Cancer Genome Anatomy Project	http://cgap.nci.nih.gov/	(14)
CGC	The Cancer Gene Census	http://cancer.sanger.ac.uk/cancergenome/projects/census/	(33)
CGED	Cancer Gene Expression Database	http://cged.hgc.jp	(54)
CGWB	The Cancer Genome WorkBench	http://cgap.nci.nih.gov/cgap.html	(17)
ChromoHub V2	A database for navigators of chromatin-mediated signalling	http://www.thesgc.org/chromohub/	(49)
CID	The Cancer Immunome Database	http://ludwig-sun5.unil.ch/CancerImmunomeDB/	(68)
COSMIC	Catalogue of Somatic Mutations in Cancer	http://cancer.sanger.ac.uk/cancergenome/projects/cosmic/	(18)
COSMICMart	BioMart portal for COSMIC	https://cancer.sanger.ac.uk/cosmic/login	(32)
CTdatabase	Cancer-Testis Antigens database	http://www.cta.lncc.br/	(69)
dbDEPC 2.0	A database of Differentially Expressed Proteins in human Cancer	http://lifecenter.sgst.cn/dbdepc/index.do	(57,58)
DriverDB	Cancer Driver genes/mutations database deduced from exome-seq	http://driverdb.ym.edu.tw/DriverDB/intranet/init.do	(23)
DTP	Anti-cancer agent mechanism database	http://dtp.nci.nih.gov/docs/cancer/searches/standard_mechanism.html	(76,77)
HPtaa	The Human Potential Tumor Associated Antigen database	http://www.bioinfo.org.cn/hptaa/	(66)
IARC TP53 Database	International Agency for Research on Cancer TP53 Database	http://p53.iarc.fr/	(44,45)
ICGC Data Portal	A single entry point to ICGC	https://dcc.icgc.org/	(3)
ICPS	An Integrative Cancer Profiler System	http://server.bioicps.org/	(10)
IntOGen	Integrative Oncogenomics	http://www.intogen.org/	(80)
IntOGen Biomart	Biomart portal of IntOGen	http://biomart.intogen.org/	(83)
ITTACA	Integrated Tumor Transcriptome Array and Clinical data Analysis	http://bioinfo.curie.fr/ittaca	(53)
MethyCancer	A database of human DNA methylation and cancer	http://methycancer.psych.ac.cn/	(47)
miRCancer	miRNA-Cancer association database	http://mircancer.ecu.edu/	(50)
Mitelman Database	Database of chromosome aberrations and gene fusions in cancer	http://cgap.nci.nih.gov/Chromosomes/Mitelman	(65)
MoKCa	Mutations of Kinases in Cancer database	http://strubiol.icr.ac.uk/extra/mokca/	(59)
NCG 4.0	Network of Cancer Genes	http://ncg.kcl.ac.uk/	(84,85)
ONCOMINE	A cancer microarray database	https://www.oncomine.org/resource/login.html	(52)
OncomiRDB	A database of experimentally verified oncomiRs	http://bioinfo.au.tsinghua.edu.cn/member/jgu/oncomirdb/	(51)
PubMeth	Cancer methylation database based on text-mining of PubMed	http://matrix.ugent.be/pubmeth/	(48)
RASOnD	RAS Oncogene Database	http://www.aiims.edu/RAS.html	(20)
SCDE	The Stem Cell Discovery Engine	http://discovery.hsci.harvard.edu/	(61,62)
TCGA Roadmap	An updated road map of files in TCGA	http://tcga.github.io/Roadmap/	(9)
TGDBs	Tumor Gene Family Databases	http://www.tumor-gene.org/tgdf.html	(20)
UCSC Cancer Genomics Browser	A web-based tool for integration, visualization and analysis of cancer genomics and clinical data	https://genome-cancer.ucsc.edu/	(15,16)
UMD TP53 database	A database of TP53 mutations in human cancer	http://p53.fr/	(43)
Cancer type-specific databases			
CCDB	Cervical Cancer Database	http://crdd.osdd.net/raghava/ccdb/faq.php	(86)
curatedOvarianData	Clinically annotated data for the ovarian cancer transcriptome	http://bcb.dfci.harvard.edu/ovariancancer/	(89)
DDPC	Dragon Database of Genes associated with Prostate Cancer	http://cbrc.kaust.edu.sa/ddpc/index.php	(88)
G2SBC	Genes-to-Systems Breast Cancer database	http://www.itb.cnr.it/breastcancer	
HLungDB	Human Lung Cancer Database	http://www.megabionet.org/bio/hlung/	(92)
Osteosarcoma Database	Database of Osteosarcoma-associated genes and microRNAs	http://osteosarcoma-db.uni-muenster.de/	(93)
PED	Pancreatic Expression Database	http://www.pancreasexpression.org/	(94)
RCDB	Renal Cancer gene Database	http://www.juit.ac.in/attachments/jsr/rcdb/homenew.html	(95)

Underlined denote abbreviated form. SNP, single-nucleotide polymorphism; CNA, copy-number alterations; exome-seq, exome-sequencing.
